# Temporal Bone Pneumatization: Relationship With Sex and Variants of the Ethmoid and Sphenoid Bone

**DOI:** 10.1097/SCS.0000000000007809

**Published:** 2021-07-07

**Authors:** Daniele Gibelli, Michaela Cellina, Stefano Gibelli, Marta Panzeri, Giovanni Termine, Chiara Floridi, Chiarella Sforza

**Affiliations:** ∗ Department of Biomedical Sciences for Health, University of Milan; † Department of Radiology, Fatebenefratelli Hospital; ‡ Department of Otorhinolaryngology, Fatebenefratelli Hospital, ASST Fatebenefratelli Sacco; § Department of Diagnostic Radiology, San Raffaele Hospital, Milan; || Department of Radiology, University Hospital “Umberto I – Lancisi – Salesi”, Ancona, Italy.

**Keywords:** Anatomy, ethmoid bone, sphenoid sinus, temporal bone

## Abstract

Pneumatization variants of the temporal bone have a crucial importance in several surgical interventions. However, very few data are known about possible correlation with other pneumatization variants.

Pneumatization of glenoid fossa, petrous apex, and infralabyrinthine portion of temporal bone was assessed in 200 computed tomography scans, equally divided between males and females (18–92 years). Pneumatization variants of the ethmoid (concha bullosa, agger nasi, pneumatized crista galli) and the sphenoid sinuses (pneumatized pterygoid processes, anterior clinoid processes, dorsum sellae, volume) were recorded as well.

Differences in prevalence of each pneumatization type according to sex and side, among different portions of the temporal bone, and between temporal bone and the pneumatized variants of the ethmoid bone and sphenoid sinuses, were assessed through chi-square test (*P* < 0.05). Differences in sphenoid volume among different pneumatization degrees of the temporal bone were assessed through 1-way analysis of covariance test (*P* < 0.05).

Pneumatization of the petrous apex and the infralabyrinthine portion was significantly more frequent in males than in females, whereas pneumatization of the glenoid fossa was more often observed on the right side (*P* < 0.05). Variants of the temporal bone are all related one with each other in males, whereas in females only the relationship between pneumatized petrous apex and infralabyrinthine portion was found. Moreover, in females the pneumatized petrous apex was related with pneumatized anterior clinoid process, and the pneumatized infralabyrinthine portion was related with the sphenoid sinus volume.

Results may be useful for predicting these important variants in planning surgical interventions of the cranial base.

## Abstract 2

Supplemental Digital Content is available in the text

Pneumatization represents the development of air-filled cavities within bones: they usually include paranasal sinuses (frontal, maxillary, and sphenoid sinuses), air cells within the bodies of the ethmoid bone, and the mastoid process of the temporal bone.^1^,^2^ However, other portions of the temporal bone may be affected by pneumatization, including the articular process and the glenoid fossa,^3^ the petrous bone,^4^ and the infralabyrinthine portion.^5^ These variants have a special importance in clinical and surgical contexts: generally, air cavities represent areas of minimal resistance against the spread of several pathologies within the temporal bone, such as otitis media and cholesteatoma.^5^ For what concerns the glenoid fossa, pneumatization renders it more fragile and represents a potential contraindication in surgical intervention involving the temporomandibular joint, such as the articular eminectomy.^6^ Moreover, the existence of pneumatized areas near the structures included in the petrous portion of the temporal bone (internal carotid artery, vestibulocochlear nerve, facial nerve, vidian nerve) are considered possible complicating factors during surgical procedures focusing on these targets.^4^ This risk is confirmed by the increased incidence of cerebrospinal fluid fistula after cranial base surgery in patients affected by enlarged pneumatization of the temporal bone.^7^,^8^

On the other hand, the infralabyrinthine approach is described in literature as a surgical option for the treatment of several neoplasms of the jugular foramen, internal acoustic meatus, and carotid foramen: in this case, instead, a poor pneumatization of the area represents a limitation to surgical procedure.^9^

Although the surgical implications deriving from the presence of the above-mentioned variants of pneumatization, very few articles have analyzed their prevalence, providing a fragmentary description. Even less works have focused on their possible relationship with sex where the reported data are often contradictory. Bronoosh et al^10^ and Khojastepour et al^6^ analyzed the pneumatization of the articular process and did not find any statistically significant correlation with sex. On the other side, Tan et al^11^ took the pneumatization of the mastoid process, petrous apex, and infralabyrinthine compartment into consideration, and found that only the latter 2 portions were significantly more pneumatized in males than in females, whereas the former one did not show any significant difference according to sex. Moreover, no information is given about the pneumatization of the glenoid fossa.

Finally, very few data are available about the possible correlation between different form of pneumatization of the temporal bone and other variants of pneumatized structures: Tan et al^11^ verified a positive correlation between pneumatization of the mastoid process, petrous apex, and infralabyrinthine compartment, but they did not consider the glenoid fossa; on the other side, Kim et al^12^ found a positive correlation between the volume of mastoid cells and the volume of sphenoid sinuses on 60 patients, but did not analyze other types of temporal pneumatization or other similar variants in the ethmoid bone (such as concha bullosa, agger nasi, pneumatization of crista galli) as well as in the sphenoid sinuses (pneumatized pterygoid processes, anterior clinoid processes, dorsum sellae).

The present article aims at providing an additional contribution to the analysis of pneumatized variants of the temporal bone: in detail, the correlation between all the 3 main variants (pneumatization of the glenoid fossa, petrous apex, and infralabyrinthine portion) was assessed. In addition, the correlation between the different forms of pneumatization in the temporal, ethmoid, and sphenoid bones was analyzed as well. The aim is to verify a possible link between the different pneumatization variants: results will be useful to predict potential risks of complicating factors and to improve the management of surgical treatment.

## MATERIALS AND METHODS

### Materials

Two hundred computed tomography (CT) scans, equally divided between males and females, were chosen from a hospital database: patients with pathologies of the temporal bone (cholesteatoma and otitis media) as well as malformations of the cranium or fractures of the cranial base were excluded from the study. Age ranged between 22 and 91 years (mean: 49.8 ± 18.5) for male subjects, and between 18 and 92 years for females (mean: 54.4 ± 21.7). Possible significant differences of age according to sex were assessed through Student *t*-test (*P* < 0.05).

The study follows international laws and guidelines (Helsinki Declaration) and was approved by the local ethical committee (7331/2019).

Most frequent reasons for CT-scan were screening for post-traumatic cranial fractures (57.3%), suspected sinusitis, nasal, and paranasal symptoms (20.0%) or neurological symptoms (headache, vertigo) (12.7%). All CT scans were performed on the same CT scanner, Somatom Definition Flash (Siemens, Forchheim, Germany), following these acquisition parameters: kV: 120, mAs: 320, collimation: 40 × 0.6 mm, tube rotation: 1 second; reconstruction thickness: 3 mm. All CT datasets were reconstructed on axial, coronal, and sagittal planes with reconstruction filters set at H21s smooth for soft tissues and H60 sharp for bone tissue.

### Analysis of Pneumatization Variants

**FIGURE 1:**
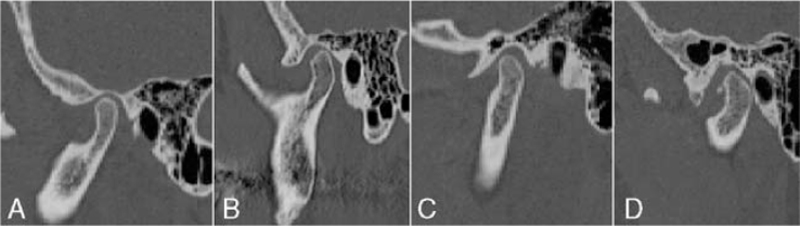
Types of pneumatization of the glenoid fossa according to the classification by Al Faleh and Ekram^1^: (A) type 1: pneumatization limited to the mastoid process; (B) type 2: pneumatization extending between the mastoid process and the mandibular fossa; (C) type 3: pneumatization extending between the deepest point of the mandibular fossa and the tip of the articular eminence; (D) type 4: pneumatization exceeding beyond the crest of the articular eminence.

On each CT scan the pneumatization of the glenoid fossa, the petrous apex and the infralabyrinthine portion of the temporal bone was assessed by 2 authors, an anatomist and a radiologist with decennial experience in radiological anatomy. Pneumatization of the glenoid fossa was assessed through the classification by Al Faleh and Ibrahim,^11^ as follows:

- Type 1: pneumatization limited to the mastoid process;
- Type 2: pneumatization extending between the mastoid process and the mandibular fossa;
- Type 3: pneumatization extending between the deepest point of the mandibular fossa and the tip of the articular eminence;
- Type 4: pneumatization exceeding beyond the crest of the articular eminence (Fig. 1).

**FIGURE 2:**
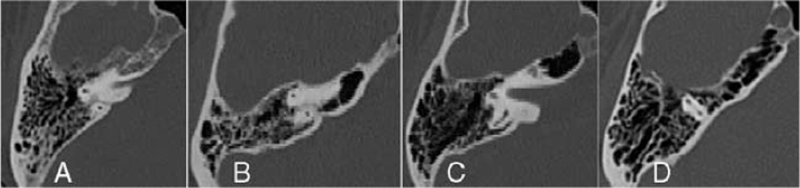
Types of pneumatization of the petrous apex according to the classification by Han et al^6^: (A) type 1: no air cells are present close to the inner ear; (B) type 2: less than half of the petrous apex medially to the labyrinth is pneumatized; (C) type 3: more than half of the petrous apex medially to the labyrinth is pneumatized; (D) type 4: most of the petrous apex area medially to the labyrinth is composed by air cells.

For the petrous apex, transversal sections were analyzed, and the classification suggested by Han et al^13^ was applied, as described below:

- Type 1: no air cells are present close to the inner ear;
- Type 2: less than half of the petrous apex medially to the labyrinth is pneumatized;
- Type 3: more than half of the petrous apex medially to the labyrinth is pneumatized;
- Type 4: most of the petrous apex area medially to the labyrinth is composed by air cells (Fig. 2).

**FIGURE 3:**
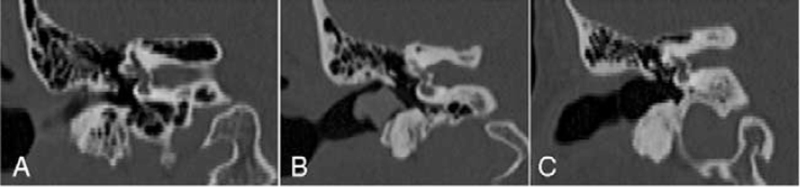
Types of pneumatization of the infralabyrinthine portion according to the classification by Marchioni et al^12^: (A) presence of well-pneumatized bone at the most inferior and medial portion of the temporal bone below the internal acoustic meatus; (B) limited pneumatized bone below the labyrinth; (C) absence of air cells at the most inferior and medial portion of the temporal bone below the internal acoustic meatus.

The pneumatization of the infralabyrinthine portion was assessed in sagittal view through the classification by Marchioni et al,^14^ as follows:

- Type A: presence of well-pneumatized bone at the most inferior and medial portion of the temporal bone below the internal acoustic meatus;
- Type B: limited pneumatized bone below the labyrinth;
- Type C: absence of air cells at the most inferior and medial portion of the temporal bone below the internal acoustic meatus (Fig. 3).

**FIGURE 4:**
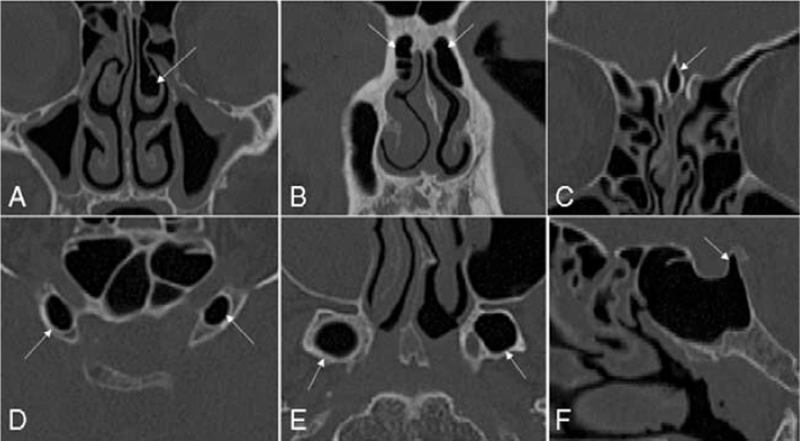
Pneumatization variants of the ethmoid bone (A–C) and the sphenoid sinuses (D–F): (A) concha bullosa; (B) agger nasi; (C) pneumatized crista galli; (D) pneumatized anterior clinoid processes; (E) pneumatized pterygoid processes; (F) pneumatized dorsum sellae. Variants are indicated by the white arrows.

Moreover, the presence of pneumatization variants of the ethmoid bone, including concha bullosa, agger nasi, pneumatized crista galli was recorded as well (Fig. 4).

Finally, the pneumatization characteristics of the sphenoid sinuses were analyzed: more precisely, for each CT-scan a three-dimensional model of sphenoid sinuses was extracted through a semi-automatic segmentation (ITK-SNAP software, University of Pennsylvania, Philadelphia, PA),^15^ and its volume was automatic calculated by the three-dimensional elaboration VAM software (Canfield Scientific, NJ [Parsippany, NJ (USA)]). In addition, pneumatization variants (pneumatized pterygoid processes, anterior clinoid processes, and dorsum sellae) were recorded (Fig. 4).

### Statistical Analyses

Possible differences in prevalence of each pneumatization type of temporal bone according to sex and side were assessed through chi-square test (*P* < 0.05).

Possible correlation among the pneumatization degree of different portions of the temporal bone (glenoid fossa, petrous apex, and infralabyrinthine portion), and between temporal bone and the pneumatized variants of the ethmoid and sphenoid bone (concha bullosa, agger nasi, pneumatized crista galli; pneumatized pterygoid process, anterior clinoid process and dorsum sellae) were assessed through chi-square test (*P* < 0.01), separately for males and females and for the right and left side.

Significant differences in sphenoid volume among different pneumatization degrees of the temporal bone were assessed through 1-way analysis of covariance test (*P* < 0.05), separately for males and females and for the right and left side. In case of statistically significant differences, post hoc Tukey test was used, applying Bonferroni correction (*P* < 0.01).

## RESULTS

No statistically significant differences for age were found according to sex (*P* > 0.05).

Results about the pneumatization degree of each portion of the temporal bone are shown in Supplementary Digital Content, Table 1, http://links.lww.com/SCS/C792: generally, pneumatization was found between 29% and 40% of cases in the glenoid fossa, between 20% and 32% in the petrous apex and between 20% and 40% in the infralabyrinthine portion of the temporal bone. Significant differences in pneumatization distribution between males and females were found for the petrous apex and the infralabyrinthine portion, showing the males a higher percentage of pneumatization than the females on both the right and left side (*P* < 0.05). On the other hand, pneumatization of the glenoid fossa showed significant differences according to side, being more often observed on the right side, in both males and females (*P* < 0.05).

Moreover, statistical analyses showed that pneumatization degrees of different portions of the temporal bone are all related one with each other in males (*P* < 0.05); on the other hand, in females the pneumatized petrous apex was significantly related with the infralabyrinthine portion, whereas the petrous apex was related with the glenoid fossa, but only on the right side (Supplementary Digital Content, Table 2, http://links.lww.com/SCS/C792 and Supplementary Digital Content, Table 3, http://links.lww.com/SCS/C792).

Supplementary Digital Content, Table 4, http://links.lww.com/SCS/C792 shows the prevalence of each variant of pneumatization of the ethmoid and sphenoid bones: the most frequent variant was concha bullosa (between 57% and 64%), followed by pneumatized pterygoid processes (between 41% and 52%) and agger nasi (between 41% and 51%). Generally, males showed a positive correlation between pneumatization of the petrous apex and the pneumatized crista galli, but only on the left side (*P* < 0.01, Supplementary Digital Content, Table 5, http://links.lww.com/SCS/C792). In females the pneumatized petrous apex was positively related with pneumatized anterior clinoid processes; moreover, the pneumatized infralabyrinthine portion was related with the sphenoid sinus volume, on both the right and left side (*P* < 0.01). In addition, the infralabyrinthine portion was positively related with the pneumatized anterior clinoid process, but only on the left side (*P* < 0.05, Supplementary Digital Content, Table 6, http://links.lww.com/SCS/C792).

## DISCUSSION

Very few information is available about the pneumatization of the temporal bone. Air cells of the temporal bone are reported to pneumatize after puberty and reach the final size in the adulthood.^16^ However, literature has reported cases of pneumatized temporal bone between 7 and 10 years.^9^ Even less is known about the factors determining pneumatization of bones, although literature agree in recognizing a combination of genetic and environmental factors, similarly to the development of other paranasal sinuses.^17^ From this point of view, the assessment of possible correlation between pneumatized forms of temporal bone and other pneumatized structures may provide more information about these variants.

The present study provides novel information for increasing our knowledge about pneumatization of the temporal bone: first of all, in the chosen sample pneumatized forms are generally less common than the not pneumatized variant: among all the forms, pneumatized glenoid fossa was most frequently observed (29%–56%). Unfortunately, very few data are available in literature to compare with the present results: Khojastepour et al^6^ reported prevalence of type 2 pneumatization of the mandibular fossa in both males and females (37.3% and 32.9%, respectively) in an Iranian sample.^6^ Moreover, they excluded sexual dimorphism of this variant, as also confirmed by the present results. Conversely, type 4 is rarely reported, with a prevalence of 5.1% in males and 3.1% in females, similarly to the present study.

On the other side, the petrous apex and the infralabyrinthine portion are less frequently pneumatized: interestingly, for the former one, Tan et al^11^ in sample from Singapore reported a prevalence of type 2 for pneumatization (54.8%), which may suggest a possible ethnic variability of these variants. Moreover, they confirmed the sexual dimorphism of pneumatization of the petrous apex and the infralabyrinthine portion of the temporal bone, more pronounced in males than in females.^10^ This result was found also by the present article and may explain the higher risk of Cerebrospinal fluid fistula in females than in males, reported by literature.^8^

A novel result concerns the prevalence of the pneumatized glenoid fossa on the right side than on the left side, in both sexes: this information has not been reported by literature so far, but it is worth being explored in depth, as it suggests an asymmetrical development of pneumatized areas in this part of the temporal bone. A hypothesis may involve the function and asymmetric movement of temporomandibular joint: further studies are needed to explore this possible relationship.

Another interesting result concerns the correlation of various forms of pneumatization: all the pneumatized variants of the temporal bone are in positive correlation one with each other in males, whereas in females the pneumatized petrous apex was significantly related with the infralabyrinthine portion only. These results may suggest a different development of pneumatized areas of temporal bone according to sex.

The possible different development of pneumatization in males and females may be suggested also by the different correlation found with pneumatization variants of the ethmoid bone and sphenoid sinuses: whereas in males no important relationship was found, in females the pneumatized petrous apex was positively related with pneumatized anterior clinoid process, and the pneumatized infralabyrinthine portion with the sphenoid sinus volume, on both sides (*P* < 0.01). This information is novel, as so far literature has only verified a relationship between the degree of pneumatization of the mastoid process and the sphenoid sinuses, without considering other areas of temporal bone.^4^,^18^

The present article highlighted a strict correlation between different forms of pneumatization, in temporal, ethmoid, and sphenoid bone: in addition, the prevalence and sexual dimorphism of different variants were reported. Results will be used to predict the possible surgical risk of patients caused to their pneumatization profile. Moreover, differences in glenoid fossa pneumatization according to side suggest to explore the possible pneumatization of temporal bone on both sides in the presurgical phase. The assessment of pneumatization variants of the temporal bone will be fundamental in planning surgical procedures involving the glenoid fossa, the petrous apex, and the infralabyrinthine portion.

In conclusion, the present article brings about novel data about the pneumatization of different areas of temporal bone and possible correlation with other pneumatization variants. Results will provide novel hints for the next research in this field.

## Supplementary Material

SUPPLEMENTARY MATERIAL
