# Supplementary material for: Temporal Bone Pneumatization: Relationship With Sex and Variants of the Ethmoid and Sphenoid Bone
Source: J Craniofac Surg. 2021 Jul 7;32(8):2888–91. doi: 10.1097/SCS.0000000000007809 (PMC10292571; doi:10.1097/SCS.0000000000007809)
Supplement: SUPPLEMENTARY MATERIAL [file jcrsu-32-2888-s001.docx]

| **Right side** |  | | **Glenoid fossa** | | **Petrous apex** | |
| --- | --- | --- | --- | --- | --- | --- |
|  | **Type** | | **Males** | **Females** | **Males** | **Females** |
|  | **1** | | 60 | 71 | 69 | 80 |
|  | **2** | | 27 | 23 | 10 | 14 |
|  | **3** | | 6 | 2 | 13 | 4 |
|  | **4** | | 7 | 4 | 8 | 2 |
|  | **Sex** | **Chi square test** | 4.062 | | 9.843 | |
|  |  | **p** | 0.255 | | 0.020* | |
| **Left side** |  | | **Glenoid fossa** | | **Petrous apex** | |
|  | **Type** | | **Males** | **Females** | **Males** | **Females** |
|  | **1** | | 44 | 58 | 68 | 81 |
|  | **2** | | 25 | 21 | 11 | 13 |
|  | **3** | | 15 | 6 | 11 | 4 |
|  | **4** | | 16 | 15 | 10 | 2 |
|  | **Sex** | **Chi-square test** | 6.159 | | 9.901 | |
|  |  | **p** | 0.104 | | 0.019* | |
|  | | | | | | |
| **Side** | | **Chi square test** | 9.917 | 9.769 | 0.444 | 0.043 |
|  |  | **p** | 0.019* | 0.021* | 0.931 | 0.998 |

Supplemental Table 1: prevalence of different degrees of pneumatisation observed in glenoid fossa and petrous apex (unit: percentage), and results of Chi-square test for testing differences according to sex and side (*: p<0.05)

| **Right side** | **Type** | | **Males** | | **Females** |
| --- | --- | --- | --- | --- | --- |
|  | A | | 21 | | 6 |
|  | B | | 19 | | 17 |
|  | C | | 60 | | 77 |
|  | - | | - | | - |
|  | **Sex** | **Chi square test** | 10.554 | | |
|  |  | **P** | 0.005* | | |
| **Left side** | **Type** | | **Males** | | **Females** |
|  | A | | 22 | | 6 |
|  | B | | 15 | | 14 |
|  | C | | 63 | | 80 |
|  | - | | - | | - |
|  | **Sex** | **Chi-square test** | 11.198 | | |
|  |  | **p** | 0.004* | | |
|  | | | | | |
| **Side** | | **Chi square test** | 0.567 | 0.348 | |
|  |  | **p** | 0.753 | 0.840 | |

Supplemental Table 2: prevalence of different degrees of pneumatisation observed in infralabyrinthine portion (unit: percentage), and results of Chi-square test for testing differences according to sex and side (*: p<0.05)

|  | Males | | Females | |
| --- | --- | --- | --- | --- |
|  | Right | Left | Right | Left |
| Concha bullosa (%) | 59 | 64 | 57 | 62 |
| Agger nasi (%) | 50 | 41 | 51 | 47 |
| Pneumatized crista galli (%) | 10 | | 14 | |
| Pneumatised pterygoid process (%) | 41 | 44 | 49 | 52 |
| Pneumatised anterior clinoid process (%) | 23 | 16 | 24 | 23 |
| Pneumatised dorsum sellae (%) | 41 | | 48 | |
| Sphenoid sinus volume (cm^3^) | 4.9±3.4 | 5.1±3.2 | 4.3±2.6 | 4.3±2.4 |

Supplemental Table 3: prevalence of pneumatisation variants of the ethmoid and sphenoid bones

| **Males** | | | |
| --- | --- | --- | --- |
| Right side  Left side | Glenoid fossa | Petrous apex | Infralabyrinthine portion |
| Glenoid fossa |  | 45.734  (<0.001*) | 21.886  (0.001*) |
| Petrous apex | 39.328  (<0.001*) |  | 51.293  (<0.001*) |
| Infralabyrinthine portion | 25.411  (<0.001*) | 54.748  (<0.001*) |  |
| **Females** | | | |
| Right side  Left side | Glenoid fossa | Petrous apex | Infralabyrinthine portion |
| Glenoid fossa |  | 21.696  (0.001*) | 8.555  (0.200) |
| Petrous apex | 11.919  (0.218) |  | 41.361  (<0.001*) |
| Infralabyrinthine portion | 4.879  (0.559) | 49.096  (<0.001*) |  |

Supplemental Table 4: relationship between pneumatisation degrees of different portions of the temporal bone (Chi-square test, *: p<0.05)

| Males | | Glenoid fossa | | Petrous apex | | Infralabyrinthine portion | |
| --- | --- | --- | --- | --- | --- | --- | --- |
|  |  | Right | Left | Right | Left | Right | Left |
| Chi square test | Concha bullosa | 0.236  (0.972) | 0.305  (0.959) | 2.379  (0.498) | 1.556  (0.669) | 0.840  (0.657) | 3.946  (0.139) |
|  | Agger nasi | 3.971  (0.265) | 1.447  (0.695) | 1.607  (0.658) | 0.764  (0.858) | 2.731  (0.255) | 1.649  (0.438) |
|  | Pneumatized crista galli | 4.785  (0.188) | 3.708  (0.295) | 10.696  (0.013) | 12.109  (0.007*) | 2.657  (0.265) | 2.755  (0.252) |
|  | Pneumatised pterygoid process | 2.740  (0.434) | 5.874  (0.118) | 2.271  (0.518) | 7.661  (0.054) | 3.981  (0.137) | 4.432  (0.109) |
|  | Pneumatised anterior clinoid process | 1.459  (0.692) | 0.650 (0.885) | 3.303  (0.347) | 1.973  (0.578) | 6.369  (0.041) | 5.296  (0.071) |
|  | Pneumatised dorsum sellae | 0.925  (0.819) | 4.443  (0.217) | 2.212  (0.530) | 2.177  (0.536) | 1.600  (0.449) | 2.200  (0.333) |
| One-way ANCOVA test | Sphenoid sinus volume | 0.680  (0.566) | 1.402  (0.247) | 1.581  (0.199) | 2.134  (0.101) | 0.680  (0.566) | 1.449  (0.240) |

Supplemental Table 5: relationship between pneumatisation degrees of different portions of the temporal bone and pneumatisation variants of the ethmoid and sphenoid bone in males (*: p<0.05)

| Females | | Glenoid fossa | | Petrous apex | | Infralabyrinthine portion | |
| --- | --- | --- | --- | --- | --- | --- | --- |
|  |  | Right | Left | Right | Left | Right | Left |
| Chi square test | Concha bullosa | 5.612  (0.132) | 8.394  (0.039) | 3.042  (0.385) | 0.400  (0.940) | 0.299  (0.861) | 0.720  (0.698) |
|  | Agger nasi | 1.174  (0.756) | 3.588  (0.310) | 0.246  (0.970) | 0.831  (0.842) | 3.896  (0.143) | 2.939  (0.230) |
|  | Pneumatized crista galli | 1.197  (0.754) | 1.572  (0.666) | 3.595  (0.309) | 1.878  (0.598) | 1.126  (0.569) | 2.651  (0.266) |
|  | Pneumatised pterygoid process | 2.131  (0.546) | 2.778  (0.427) | 3.011  (0.390) | 2.031  (0.566) | 0.803  (0.669) | 3.856  (0.145) |
|  | Pneumatised anterior clinoid process | 3.459  (0.326) | 1.807  (0.613) | 13.377  (0.004*) | 13.722  (0.003*) | 6.428  (0.040*) | 13.944  (0.001*) |
|  | Pneumatised dorsum sellae | 8.544  (0.036) | 2.730  (0.435) | 3.646  (0.302) | 2.526  (0.471) | 7.647  (0.022) | 7.445  (0.024) |
| One-way ANCOVA test | Sphenoid sinus volume | 1.842  (0.145) | 1.135  (0.339) | 1.959  (0.125) | 1.946  (0.127) | 5.986  (0.004*) | 5.722  (0.004*) |

Supplemental Table 6: relationship between pneumatisation degrees of different portions of the temporal bone and pneumatisation variants of the ethmoid and sphenoid bone in females (*: p<0.05)
